# Maternal lipids in pregnancy are associated with increased offspring cortisol reactivity in childhood

**DOI:** 10.1016/j.psyneuen.2017.04.018

**Published:** 2017-09

**Authors:** Theresia H. Mina, Marius Lahti, Amanda J. Drake, Shareen Forbes, Fiona C. Denison, Katri Räikkönen, Jane E. Norman, Rebecca M. Reynolds

**Affiliations:** a University/BHF Centre for Cardiovascular Science, Queen’s Medical Research Institute, University of Edinburgh, 47 Little France Crescent, Edinburgh EH16 4TJ, Scotland, UK; b Department of Psychology and Logopedics, Faculty of Medicine, University of Helsinki, 00014 Helsinki, Finland; c MRC Centre for Reproductive Health, Queen’s Medical Research Institute, University of Edinburgh, Edinburgh EH16 4TJ, Scotland, UK; d Tommy’s Centre for Maternal and Fetal Health, Queen’s Medical Research Institute, University of Edinburgh, Edinburgh EH16 4TJ, Scotland, UK

**Keywords:** Prenatal, Glucose, Lipid, Obesity, Child, Cortisol

## Abstract

Prenatal programming of hypothalamic-pituitary-adrenal (HPA) axis activity has long term implications for offspring health. Biological mechanisms underlying programming of the offspring HPA axis are poorly understood. We hypothesised that altered maternal metabolism including higher maternal obesity, glucose and lipids are novel programming factors for altered offspring HPA axis activity. Salivary cortisol levels were measured in 54 children aged 3–5 years under experimental conditions (before and after a delay of self-gratification test). Associations of child cortisol responses with maternal obesity in early pregnancy and with fasting glucose, triglycerides, HDL and total cholesterol measured in each pregnancy trimester were tested. Higher levels of maternal triglycerides and total cholesterol throughout pregnancy were associated with increased offspring cortisol reactivity. The associations were independent of maternal obesity and other confounders, suggesting that exposure to maternal lipids could be a biological mechanism of *in utero* programming of the offspring’s HPA axis.

## Highlights

• We show that the maternal prenatal metabolic milieu predicts a child’s cortisol response to a stressor at 3–5 years of age.
• Increased maternal prenatal obesity, triglycerides and total cholesterol were associated with increased offspring cortisol reactivity.
• Maternal metabolism may be a novel programming factor for the offspring’s HPA axis.

## 1 Introduction

Early life programming of the hypothalamic pituitary adrenal (HPA) axis is one of the key mechanisms proposed to link prenatal development with later life disease (Reynolds, 2013). Increased offspring HPA axis activity is associated with an adverse *in utero* environment and is observed at birth (Gitau et al., 2004), and persists through adulthood (Reynolds et al., 2001, van Montfoort et al., 2005). Altered HPA axis activity is associated with a range of health problems including an increased risk of metabolic disease, psychiatric symptoms, and cognitive decline (Chrousos, 2009, Constantinof et al., 2016). Mechanisms underlying the early life programming of the HPA axis remain poorly understood.

The evidence that physical and psychological adversity in prenatal or early postnatal life associates with altered child’s HPA axis activity has been systematically reviewed (Hunter et al., 2011). Yet few studies have considered biological mechanisms such as whether maternal metabolic status during pregnancy could alter the offspring HPA axis. In one study higher maternal body mass index (BMI) during pregnancy was not associated with salivary cortisol diurnal rhythm or stress reactivity in preschool children (Elhassan et al., 2015), but in another higher maternal central obesity during pregnancy predicted higher fasting plasma cortisol levels in 8.5 years-old children (Phillips et al., 2005). In children exposed to maternal gestational diabetes, there were no salivary cortisol differences following the Trier Social Stress Test for children (TSST-c) at age 13 (Krishnaveni et al., 2015). To our knowledge no studies have considered whether *in utero* exposure to other maternal metabolites, including lipids, influences the HPA axis responses in younger children.

We hypothesised that increased prenatal exposure to an adverse maternal metabolic milieu would be associated with altered child cortisol reactivity under experimental conditions. To test this hypothesis we studied a cohort of children born to mothers with very severe obesity (SO, BMI ≥40 kg/m^2^ at first antenatal visit) and lean controls (BMI ≤ 25 kg/m^2^) in whom maternal metabolic profiles, including glucose and lipids had been characterized at gestational weeks 17, 28 and 36 (Forbes et al., 2015, Mina et al., 2015). Previously we reported that mothers with SO had higher glucose and lipids levels than the lean (Forbes et al., 2015), thereby providing a sample with variability among the maternal metabolic markers of interest. The case-control design of this larger cohort (SO vs. lean) also enabled us to examine the consequences of exposure to maternal SO compared with lean on child cortisol reactivity. In the current study, we characterized children’s HPA axis responses by measuring salivary cortisol profiles under experimental conditions before and after a test of delay of self-gratification.

## 2 Methods

### 2.1 Participants

Participants were children aged 3–5 years born to SO or lean mothers who were taking part in a follow-up study of the consequences of exposure to obesity in pregnancy in Midlothian, Scotland (Forbes et al., 2015, Mina et al., 2015). Ethical approval (REC: 14/WS/1046, R&D: 2014/0278) and written informed consent were obtained from all participants. The protocol for the follow-up study has been previously described (Mina et al., 2017). We collected saliva samples for the measurement of cortisol levels from 79 term-born children under ‘experimental’ conditions i.e. before and after a test of self-gratification, the ‘Marshmallow Test’. Eight sample sets were incomplete and 17 could not be linked to maternal data, leaving 54 salivary cortisol profile datasets (31 lean and 23 SO) for analysis.

### 2.2 Saliva sample collections for cortisol measurement

Mothers were asked not to give their child drinks or food for at least half an hour before the appointment at the Wellcome Trust Clinical Research Facility for Children, the Royal Hospital for Sick Children, Edinburgh. Children’s saliva samples were collected before and after the Marshmallow Test, a test of delayed self-gratification (Mischel et al., 1989), which shows predictive validity on cognitive and behavioral outcomes in child- and adulthood (Mischel et al., 1989, Casey et al., 2011). Briefly, the children were presented with a single marshmallow and instructed that they may eat it immediately, but if they waited for 15 min, they would receive a second marshmallow as a reward. Thus the collection time of the after-test saliva samples ranged between 0 and 15 min depending on the test outcome. Marshmallows do not interfere with salivary cortisol levels (Clements et al., 2007), but the second saliva sample was always collected before any marshmallow reward was given. Children’s saliva samples were collected using SalivaBio Children’s Swab set (Salimetrics, Suffolk, UK) as per the manufacturer’s protocol, extracted from the cotton swab by centrifuging the tube at 3500 rpm (1500 g) for 15 min at 4 °C, and frozen at −80 °C.

### 2.3 Laboratory analyses

Salivary cortisol levels were measured by Enzyme-Linked Immuno- abSorbent Assay (ELISA) using an expanded-range-high-sensitivity salivary cortisol enzyme immunoassay kit (Salimetrics^®^, Pasadena, USA) as per the manufacturer’s protocol. Samples were run such that each 96-well plate contained randomly selected sample sets of children of both lean and SO mothers in duplicates. Plates were read at 450 and 490 nm by the Optimax tunable microplate reader using Softmax Pro 4.8 (Molecular Devices, Sunnyvale, USA).

The sample reading (B) = [reading at 450 nm] − [reading at 490 nm] − [mean reading from non-specific binding wells]. The values (B) were then divided by mean values of standard zero (Bo). The B/Bo sample values were fitted to a standard curve using Log (inhibitor) vs. response- variable slope (four parameters) of dose-response inhibition (Graphpad Prism 6, La Jolla, USA). When salivary cortisol levels were found to be >3 μg/dl (maximal end-point of the standard curve), they were diluted using the diluent (Salimetrics^®^) as per the manufacturer’s protocol. When the salivary cortisol levels were <0.012 μg/dl (minimal end-point of the standard curve), they were assumed to be = 0.012 μg/dl. Intra-assay coefficient of variation (CV) was 3.7% (all samples) and 4.7% (standard only) as compared to the manufacturer’s 4.6%. The inter-assay CV was 4.6% as compared to the manufacturer’s 6.0%.

### 2.4 Characterizing children’s salivary cortisol profile under experimental conditions

We quantified 3 output variables, including: 1) Δ = cortisol after test − cortisol before the test; 2) Area-under-the-curve- by increase (***AUC_i_***) = [Δ * Δ time]/2; 3) steepness *=* Δ cortisol/Δ time, where Δ time represents the duration of the self-gratification delay. ***AUC_i_*** indicates changes in salivary cortisol levels over the course of time, whereas steepness implies the individual cortisol reactivity to the experimental conditions.

### 2.5 Measurement of maternal prenatal metabolic markers

Maternal blood was sampled at 9 AM following an overnight fast three times during pregnancy as previously described (Forbes et al., 2015, Mina et al., 2015). Glucose levels were measured with a hexokinase-based assay and lipid components including triglycerides, HDL and total cholesterol with a colorimetric assay (Forbes et al., 2015).

### 2.6 Covariates and statistical analysis

We considered a number of potential covariates. The following covariates were excluded as the n < 10 and/or they were not significantly associated with dependent variables: gestational diabetes, maternal symptoms of prenatal and concurrent psychiatric distress, maternal smoking, level of socio-economic deprivation, time of saliva collection, seasonal variability. The final regression model included as covariates: maternal obesity status, child’s age at visit (in months), sex, parity and child birthweight obtained from hospital records.

Statistical analyses were performed using SPSS 23.0 and α = 0.05. The averaged levels of maternal metabolic measures across pregnancy were calculated. All independent and dependent variables were standardized; children’s cortisol variables were rank-normalised using Blom’s formula. Student’s *t*-tests (for continuous variables) and chi-square tests (for categorical variables) were used to compare the covariates and dependent variables between SO and lean groups. Student’s *t*-tests were applied to determine the associations of the covariates with the dependent variables, and to compare the levels of salivary cortisol according to the completion status of the Marshmallow Test. Paired Student’s *t*-test was used to compare the raw levels of salivary cortisol before and after the Marshmallow Test.

Linear regression analyses were used to determine the associations between the predictors including maternal SO status and the averaged levels of prenatal metabolic measures and the 3 output variables of the child’s cortisol profile. In the regression model of prenatal metabolic milieu, we adjusted for child’s sex, age, maternal SO status, parity and child’s birthweight. False Discovery Rate (FDR) with Benjamini & Horchberg method was set at 0.1.

## 3 Results

### 3.1 Cohort demographic and variable descriptions and background associations

Compared to lean mothers, SO mothers had elevated triglycerides and lower HDL cholesterol levels during pregnancy, though glucose and total cholesterol levels were similar (Table 1**).** Children born to SO mothers had higher birthweight, and were older when enrolled into the follow-up study (Table 1**).** Table A1 describes the associations of children’s salivary cortisol profiles with the covariates.

**Table 1:** Differences of mother and child profile according to maternal obesity status among children who provided saliva samples during the Marshmallow Test and whose mothers provided blood samples for the measurement of prenatal metabolic milieu.

Mother and child descriptions		Lean (n = 31)	SO (n = 23)	All	P0	P1	n
**Mother and child demographics**							
Parity, n (%)	Nulliparous (0)	15 (48.39)	12 (52.17)	27 (50)	<0.999^a^	–	54
	≥1	16 (51.61)	11 (47.83)	27 (50)			
Maternal BMI, mean (SD)		22.47 (1.44)	43.39 (3.29)	31.56 (10.74)	<**0.001**^b^	–	54
Child’s birthweight, Kg, mean (SD)		3.54 (0.50)	3.77 (0.45)	3.64 (0.47)	0.069^b^	–	54
Infant sex, n (%)	Male	15 (48.39)	10 (43.49)	25 (46.30)	0.747^a^	–	54
	Female	16 (51.61)	13 (56.53)	29 (53.70)			
Child's age at study visit in months, mean (SD)		47.96 (6.78)	54.53 (5.74)	50.76 (7.10)	<**0.001**^b^	–	54
**Child’s salivary cortisol profile during the Marshmallow Test, mean (SD)**							
Saliva before, mg/dl		0.18 (0.11)	0.20 (0.11)	0.18 (0.11)	0.616^b^	–	54
Saliva after mg/dl		0.20 (0.11)	0.25 (0.14)	0.22 (0.12)	0.187^b^	–	54
Delay of self-gratification (seconds)		437.0 (397.26)	448.4 (408.70)	441.92 (398.45)	0.919^b^	–	53
Δ, mg/dl		0.02 (0.06)	0.05 (0.09)	0.03 (0.08)	0.202^b^	0.053	54
Steepness		−0.18 (0.81)	0.18 (1.03)	−0.03 (0.92)	0.164^b^	**0.01**	52
***AUC_i_***		−0.12 (0.93)	−0.01 (1.08)	−0.07 (0.99)	0.705^b^	0.333	53
**Maternal biomarker,mean (SD)**							
**Glucose**, mmol/L		4.27 (0.28)	4.38 (0.24)	4.32 (0.27)	0.116^b^	–	54
**Triglycerides**, mmol/L		1.75 (0.50)	2.15 (0.53)	1.92 (0.55)	**0.008**^b^	–	53
**HDL**, mmol/L		1.82 (0.29)	1.46 (0.36)	1.67 (0.36)	>**0.001**^b^	–	53
**Total Cholesterol**, mmol/L		6.00 (0.75)	5.92 (1.01)	5.97 (0.86)	0.732^b^	–	53

SO = very severe obesity. ***AUC_i_*** = Area-under-the-curve by increase, implying changes over time. Δ = delta, the difference in cortisol levels across two time-points, steepness = gradient of line created by changes in cortisol levels. Mean (SD) of the raw data were displayed. Full cohort profile of glucose, triglycerides, HDL and cholesterol is detailed in Forbes et al. (2015).

Bold text: p ≤ 0.05, underlined text: p ≤ 0.1. **P1** values were obtained from multiple linear regressions, adjusted for child’s sex and age at visit, parity, and child’s birthweight.

a Chi-square test.

b Student’s *t*-test.

### 3.2 Maternal prenatal metabolic milieu and child’s salivary cortisol profiles

**Fig. 1:**
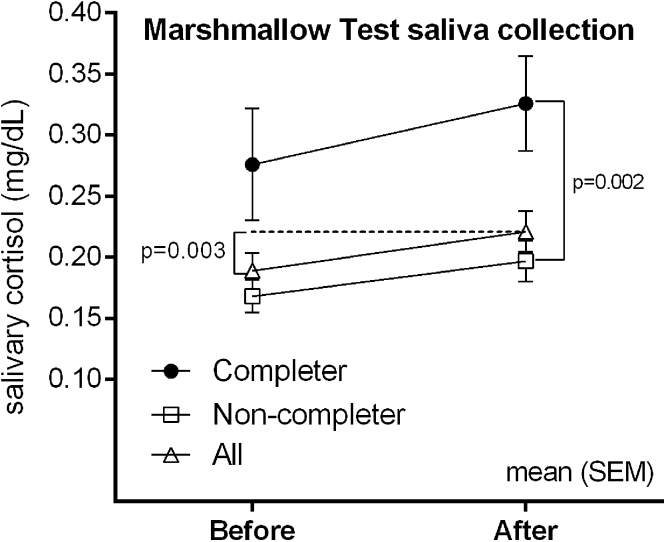
Marshmallow Test elicits cortisol stress responses in pre-schoolers. Data are Mean SEM.

In all children the average levels of salivary cortisol were higher after the Marshmallow Test than before (Mean difference _after-before_ [SD], 0.03 [0.08] μg/dl, p = 0.003, Fig. 1). Furthermore, the levels of salivary cortisol collected after the test were also higher among the 10 (4 lean, 6 SO) children who delayed gratification for 15 min as compared to those (n = 44, 26 lean, 18 SO) who ate the marshmallow before 15 min (mean [SD]_completer vs. non-completer_ = 0.33 [0.12] vs. 0.19 [0.11] μg/dl, p = 0.002, Fig. 1). There were no differences in cortisol profile variables or derivatives between children of SO and lean mothers in unadjusted analyses (Table 1). However, following adjustments for the covariates in the regression analysis, maternal SO predicted significantly higher **cortisol** steepness (cortisol reactivity) during the Marshmallow Test (Table 1).

Maternal prenatal triglyceride and total cholesterol levels were significantly correlated with higher child cortisol reactivity under experimental conditions. In the adjusted regression models (Table 2), higher averaged levels of maternal prenatal triglycerides (Δ [p = 0.004, p_Adj_ = 0.006], AUCi [p = 0.011, p_Adj_ = 0.013) and total cholesterol levels (AUCi p = 0.02, p_Adj_ = 0.0x) remained as significant predictors of increased child cortisol reactivity in experimental conditions. Neither maternal glucose or HDL levels were associated with child salivary cortisol profiles (Table 2).

**Table 2:** Children’s cortisol reactivity was associated with higher levels of maternal triglycerides and total cholesterol across pregnancy

B (95% CI), unadjusted	Glucose	Triglycerides	HDL	Total Cholesterol
Δ, μg/dl	−0.06 (-0.37, 0.26)	**0.42 (0.15, 0.69)**	0.05 (−0.24, 0.33)	**0.34 (0.00, 0.68)**
Steepness	−0.02 (-0.33, 0.30)	0.25 (-0.03, 0.54)	−0.18 (-0.46, 0.10)	−0.05 (-0.39, 0.30)
***AUC_i_***	−0.10 (0.43, 0.24)	**0.39 (0.10, 0.67)**	0.10 (−0.20, 0.41)	**0.42 (0.07, 0.77)**

B (95% CI)	Glucose	Triglycerides	HDL	Total Cholesterol
Δ, μg/dl	−0.12 (-0.44, 0.21)	**0.42 (0.14, 0.70)**	0.06 (−0.28, 0.39)	0.33 (-0.04, 0.70)
Steepness	−0.82 (-0.39, 0.22)	0.17 (−0.13, 0.47)	−0.27 (-0.58, 0.05)	−0.24 (-0.60, 0.12)
***AUC_i_***	−0.12 (-0.47, 0.24)	**0.41 (0.10, 0.72)**	0.11 (−0.26, 0.49)	**0.47 (0.08, 0.86)**

***AUC_i_*** = Area-under-the-curve by increase, implying changes over time. Δ = delta, the difference in cortisol levels across two time-points, Steepness = gradient of line created by changes in cortisol levels.

All associations were derived from the z-scores of both independent and dependent variables using multiple linear regressions. The regressions were adjusted for child’s sex and age at visit, maternal SO status, parity, and child’s birthweight.

Bold text: B with p ≤ 0.05, underlined text: p ≤ 0.1.

## 4 Discussion

Our findings of associations between higher maternal obesity, and also of higher maternal lipids independent of obesity, and greater child cortisol reactivity under experimental conditions suggest a novel biological mechanism underlying prenatal programming of the offspring HPA axis. Further, we have shown, for the first time that the Marshmallow Test a test of delay of self-gratification, evokes a cortisol response in 3–5 year old children.

The mechanisms underlying the link between associations of higher maternal triglycerides and total cholesterol levels with greater child’s cortisol reactivity under experimental conditions are unknown. In mice, prenatal exposure to a high fat diet was shown to alter gene expression levels of glucocorticoid receptor and its downstream inflammatory markers in the amygdala, resulting in heightened stress response (Sasaki et al., 2013), and slower recovery post-stress exposure (Grissom et al., 2017) suggesting programming of central HPA axis feedback sites. The lack of associations with glucose is consistent with the absence of salivary cortisol differences following the Trier Social Stress Test (TSST) in children exposed to gestational diabetes (Krishnaveni et al., 2015), suggesting that the programming of the HPA axis is metabolite-specific.

There remains debate about how best to evoke cortisol “stress responses” in young children under experimental conditions. The TSST is widely used in adult studies (Allen et al., 2014) and the modified version for children has been validated in children aged ≥7 years (Gunnar et al., 2009, Kudielka et al., 2004). However, many of the challenges trialled in preschool children do not consistently elicit increased salivary cortisol (Gunnar et al., 2009). The Marshmallow Test was not originally designed as a stress test, but the unfamiliar environment, the strange situation (mother out of sight) and the frustrating task are likely stressors for young children (Gunnar et al., 2009). In this study, we observed increased average cortisol levels after the Marshmallow Test as compared to before the test, and in children who delayed gratification until 15 min as compared to those who failed the test. Further, we found associations of lower birthweight with elevated experimental cortisol profiles, suggesting heightened stress reactivity, and consistent with other studies showing links between low birthweight and increased HPA axis activity in later life (Reynolds et al., 2001, van Montfoort et al., 2005). Therefore, we believe that salivary cortisol levels measured before and after the Marshmallow Test can be interpreted as a cortisol response to stress under experimental conditions.

The strength of this study include the prospective design with detailed biological measurements from the mother and the child. Although our sample size was small, the participants were representative of the maternal metabolic milieu of the larger cohort (Forbes et al., 2015) as no specific selection criteria were applied. Another strength is the use of a delay of gratification test as a “stress” test of cortisol reactivity. The limitations of the study include that we could not reliably examine moderation effects between maternal obesity and maternal metabolites on child cortisol reactivity due to low statistical power. Neither could we perform curvilinear analyses due to the case-control study design, nor distinguish the effect of *in utero programming* from genetic predisposition. Future studies could evaluate other stress markers from child’s saliva, such as α-amylase, and investigate whether the stressful delay of self-gratification extends to other food and beverages of different palatability independent of child’s appetite, and to non-food rewards.

## 5 Conclusions

Prenatal exposure to higher maternal lipids is associated with changes in the children’s cortisol reactivity in experimental conditions, independently of maternal obesity, and other confounders. Alterations in maternal lipids could be a novel mechanism of *in utero* programming of the offspring HPA axis.

## Conflicts of interest

None.

## Figures and Tables

**Table A1:** Background associations of children’s salivary cortisol profiles with the covariates. The components of salivary cortisol profiles were rank-normalised and converted to z-scores prior to analysis.

a Pearson's r (p) b mean difference, 95% CI, p	Child's age^a^	Raw birthweight^a^	Parity^b^	
			nulliparous vs. ≥1	
Saliva before	**0.27 (0.028)**	0.06 (0.618)	0.14 (−0.36, 0.65)	0.575
Saliva after	**0.29 (0.019)**	−0.07 (0.570)	−0.18 (-0.65, 0.31)	0.464
Δ	−0.07 (0.586)	**−0.28 (0.024)**	**−0.52 (-0.98, −0.04)**	**0.033**
Steepness	−0.18 (0.163)	**−0.27 (0.029)**	-0.48 (-0.97, 0.01)	0.054
***AUC_i_***	−0.01 (0.922)	−0.20 (0.107)	**−0.56 (-1.03, −0.09)**	**0.020**

***AUC_i_*** = Area-under-the-curve by increase, implying changes over time. Δ = delta, the difference in cortisol levels across two time-points, Steepness = gradient of line created by changes in cortisol levels.

Bold text: p ≤ 0.05, underlined text: p ≤ 0.1.

a Pearson’s r (p).

b Mean difference (95% Confidence Interval) and p.
